# Declarative and procedural learning in children and adolescents with posterior fossa tumours

**DOI:** 10.1186/1744-9081-2-9

**Published:** 2006-03-15

**Authors:** Eliana A Quintero-Gallego, Carlos M Gómez, Encarnación Vaquero Casares, Javier Márquez, Fco Javier Pérez-Santamaría

**Affiliations:** 1 Psychology Department, Neuropsychology Unity, Bosque University, Bogotá, Colombia; 2 Department of Experimental Psychology, Seville University, Seville, Spain; 3 Neurosurgery Unit, Virgen del Rocio Hospital, Seville, Spain

## Abstract

**Background:**

This quasi-experimental study was designed to assess two important learning types – procedural and declarative – in children and adolescents affected by posterior fossa tumours (astrocytoma vs. medulloblastoma), given that memory has an important impact on the child's academic achievement and personal development.

**Methods:**

We had three groups: two clinical (eighteen subjects) and one control (twelve subjects). The learning types in these groups were assessed by two experimental tasks evaluating procedural-implicit and declarative memory. A Serial Reaction-Time Task was used to measure procedural sequence learning, and the Spanish version [1] of the California Verbal Learning Test-Children's Version- CVLT- [2] to measure declarative-explicit learning. The learning capacity was assessed considering only the blocks that represent learning, and were compared with MANOVA in clinical and normal subjects. The Raven, simple reaction-time, finger-tapping test, and grooved pegboard tests were used to assess the overall functioning of subjects. The results were compared with those from a control group of the same age, and with Spanish norm-referenced tools where available

**Results:**

The results indicate the absence of procedural-implicit learning in both clinical groups, whereas declarative-explicit learning is maintained in both groups.

**Conclusion:**

The clinical groups showed a conservation of declarative learning and a clear impairment of procedural learning. The results support the role of the cerebellum in the early phase of procedural learning.

## Background

Posterior fossa tumours constitute two thirds of all paediatric brain tumours [3]. The main tumours appearing in this zone are medulloblastomas, pilocytic astrocytomas, and ependynomas, which together make up about 90% of the cases [4-6]. The most frequent are the astrocytoma and medulloblastoma. These tumours affect the cerebellum and/or its connections, so the study of cognitive and motor disturbances produced by this pathological status is important in assessing the neuropsychological profile of patients.

The principal interventions used with these tumours, and in general with most tumours, are surgery, chemotherapy, and radiotherapy. It has been demonstrated that these medical treatments cause not only motor alterations, but also deterioration in cognitive functions. Most of the relevant research has been focused on the long-term effects of radiotherapy because these are more general and severe, involving attention, memory, speed of mental processing, visuo-motor coordination [7-10], and a marked progressive impairment of overall intellectual functioning [8,11]. Given that focal irradiation of the posterior fossa includes not only the brainstem and cerebellum but also parts of the temporal lobes and other structures, overall cognitive alteration could occur. It has been found that radiotherapy can affect the neuropsychological tests of children with medulloblastoma. In addition, psychosocial functions can be affected and academic difficulties presented. All these aspects lead to a decline in the quality of life of the individuals.

It is clear that the cerebellum contributes to motor functions, coordination, and balance. However, only after the eighties has there been evidence in favour of hypotheses which relate the cerebellum to cognitive functions, such as attention [12-14], language processing [14-16], spatial abilities [16], memory [15,16], and to executive functions, such as planning and sequencing [15,17]. The relationship of the cerebellum with behavioural control and in affective modulation has also been discussed [14-16]. The conclusions regarding the contribution of the cerebellum to motor and cognitive functions come mainly from the following sources:

1. Anatomical studies: the cerebellum is connected to motor cortices, limbic areas, and polymodal associative cortex (prefrontal, parietal posterior, and superior temporal) [. e.g. [18]]. The functionality of these connections is dramatically expressed in the cerebellum-cerebral diaschisis phenomenon [19].

2. Neuroimaging studies during the performance of cognitive tasks have demonstrated a role of cerebellum in executive, attentional, perceptual, and language functions [e.g. [20]].

3. Studies related to populations with developmental disorders: populations diagnosed with hyperactivity or autism present morphometric disorders in some cerebellar structures: cerebellar vermis and/or hemispheres. When compared with control children, hyperactive children showed a decrease in the size of the posterior vermis lower portion (lobes VIII-X). In groups of autistic individuals, a decrease in the size of the vermis upper portion (lobes VI/VII) has been found [e.g. [21,22]].

4. Studies related to mutism syndrome: this syndrome is presented in some cases of children suffering posterior fossa tumours (especially those affecting the vermis) who have been intervened. This is a clear sample of cognitive deterioration (generalised) after cerebellar damage. This syndrome is manifested approximately 4 days after surgery. Patients affected by it recover completely within a few weeks or months [23,24].

5. Studies related to populations with cerebellar damage: Botez [25,26] has indicated that cerebellar lesions affect three main neuropsychological aspects: a) visuo-spatial organisation (cerebellar-parietal circuit), b) executive functions for planning and programming activities (cerebellar-frontal circuit), and c) increased RTs for visual and auditory targets, indicating a reduced speed of information processing. The latter effect is less marked in unilateral lesions.

More specifically related with the objective of the present report is the cerebellum's role in learning and memory tasks. The role of the cerebellum in motor response learning acquisition has been clearly demonstrated [27,28]. The cerebellum has a clear role in the acquisition of motor skills, in procedural learning (in close interaction with basal nuclei), and in the Pavlovian conditioning of certain motor responses (in close interaction with the hippocampus and cerebral cortex).

Specifically, a role in establishing the association between the different motor acts composing the sequence has been proposed [29]. However, an important issue is whether the deficits are due to motor impairments. Previous work has demonstrated that the participation of the cerebellum is independent of the kind of motor processes involved in accomplishing the task [30]. On the other hand, cerebellar activity during working memory tasks has been demonstrated [31], forming part of a circuit that includes prefrontal (Brodmann areas 6, 9, 44, and 46), parietal, temporal and anterior cingulated cortices, and cerebellum [32-35]. With regard to declarative memory, some authors attach importance to structures such as the cerebellum, besides the areas traditionally involved in these tasks [36].

The study's research aim was to test different types of learning task (procedural vs. declarative) in order to assess the learning potential in two clinical groups (medulloblastoma and astrocytoma tumours). Two different aspects would be considered: first, the assessment of learning and cognitive function capabilities after treatment would give some clues for establishing neuropsychological rehabilitation strategies after different types of treatment and tumour. Second, the results could give some information about the role of the cerebellum in procedural and declarative memory. In summary, this pathology could be considered an interesting model in which to study cerebellar functions and how the differential effects of certain medical therapies could also affect learning and cognitive functions.

## Methods

### Subjects

Thirty individuals participated in this study: eighteen of them were clinical patients and twelve were controls. There were two clinical groups. The first (CE: astrocytoma) comprised eleven individuals (eight girls and three boys), the mean age being 11.54 years (S.D. = 3.20). In the second group (CE+: medulloblastoma) there were seven individuals (four girls and three boys); the mean age was 13.14 years (S.D. = 1.95) at the time of the evaluation. The control group (C) comprised twelve subjects (seven girls and five boys), the mean age being 10.66 years (S.D. = 2.22). There were no significant group differences with regard to age or sex.

The subjects' mean age at the time of tumour resection was 97.00 months (S.D. = 38.25), and the age range at resection was from 23 to 130 months. The time between surgery and cognitive evaluation was from 5 to 151 months (mean= 56.94 months; S.D. = 43.38). Some of the medical variables have a high variability, but this is characteristic of our sample of patients collected in a two-year period from the Virgen del Rocio Hospital in Seville (Spain). Table 1 summarises the main characteristics of the clinical groups.

**Table 1:** Main characteristics of the clinical groups.

Histological tumor type		
• Medulloblastoma (CE+ group)	n = 7	
• Astrocitoma (CE group)	n = 11	
	**CE+ group**	**CE group**

Localization		
• Vermis	n = 7	n = 3
• Hemispheres	n = 0	n = 7
• Not information	n = 0	n = 1
Neurosurgery complications		
• No	n = 3	n = 11
• Yes	n = 4	n = 0
Chemo and radiotherapy		
• No	n = 2	n = 9
• Yes	n = 5	n = 2
Subjective deficits referred to the parents		
• No	n = 0	n = 9
• Yes	n = 7	n = 2

The members of the control group (C) were chosen from pupils of a private school in Seville. They were selected from a group of 50 children (from 6 to 16 years old) participating in a developmental-cognitive study, and who met the following criteria: standard educational opportunities, normal or corrected-to-normal visual acuity, and without any detected behavioural problems.

The present experimental protocol followed the norms of the Helsinki Declaration regarding experiments involving human subjects. The experimental protocol was approved by the ethics committee of the Virgen del Rocio Hospital.

### Motor, visuo-motor, and IQ characteristics of the subjects

Given that the clinical groups could be affected in other important behavioural characteristics, we decided to assess the motor, visuo-motor and IQ characteristics of all participants in the study. Simple motor abilities were tested with a finger-tapping task. To evaluate visuo-motor abilities, the grooved pegboard and simple reaction-time tests were used, and to assess the IQ, the Progressive Matrices of Raven were used. A brief description of the tasks follows. The general results obtained for the three groups in these tasks are presented in Table 2.

**Table 2:** Descriptive values for general performance tasks. (In brackets is shown the standard deviation)

**TEST/GROUP**	**C**	**CE**	**CE+**
RAVEN (IQ)	101,58 (10,35)	93,80 (18,56)	82,14 (12,04)
PEGBOARD (time to complete the task in seconds)	126,70 (21,05)	145,59 (41,78)	240,57 (145,99)
TAPPING (frequency in 20 seconds)	144,35 (36,08)	132,55 (18,76)	114,57 (48,19)
SIMPLE REACTION TIME (reaction time in milliseconds)	350,90 (72,57)	442,65 (142,85)	513,62 (162,51)

- Finger-tapping task. The subjects had to tap a key of the computer keyboard rapidly and repeatedly for 20 seconds with the index finger (dominant and non-dominant hand). The variable obtained was the tapping number. The mean value of tapping number in the dominant and non-dominant hand was computed. One-way ANOVA (group factor) was performed for the tapping variables. There was no statistical difference between the three groups for tapping response.

- The grooved pegboard. This consisted of a board having 25 holes with randomly positioned slots. Pegs, which had a key along one side, had to be rotated to match the hole before they could be inserted. The variable obtained from this test was the total time employed in filling all the holes using each hand. The mean value of the time needed to complete the task in the dominant and non-dominant hand was computed. Two children of the CE+ group did not end the task in the 300-second period required. The value of 300 seconds was assigned to these two subjects. One-way ANOVA (group factor) was performed for the pegboard. The effect of the group was statistically significant [F (2,27) = 5.48, p = 0.010]. The Scheffé *post hoc *test revealed that this effect was due to differences between the C and CE+ groups [p = 0.024].

- Simple Reaction Time. This task tested whether subjects were able to complete visuo-motor reaction-time tasks. A single target (a fish in the centre of the screen) was presented, and the aim of the task was to respond with the index finger as fast as possible. The target duration was 1700 ms, and in order to reduce anticipations the ISI was randomised between 1500 and 2200 ms. One-way ANOVA (group factor) was performed on the RTs. The effect of the group was statistically significant [F (2,27) = 3.720 p = 0.038]. The *post hoc *test revealed that this effect was due to differences between the C and CE+ groups (p = 0.043).

- A standard form of the Progressive Matrices of Raven was used. The score for each child was obtained taking into consideration the age of the child. The effect of the factor group was statistically significant [F (2,27 = 4.11, p = 0.028]. *Post hoc *comparisons indicated that this result was due to a significant difference between groups C and CE+ (p = 0.010).

The overall assessment indicated that there were no obvious motor, visuo-motor, or cognitive differences between the C and CE groups. The main differences were obtained between C and CE+ groups, while the CE group presented an intermediate status for all the performed tests. In any case, the obtained level of performance of the subjects was enough to participate and to complete the learning tests.

There are two possible interpretations of the differences between CE and CE+ groups described previously. First, there have been many reports that support general deficits associated to unspecific cortical and subcortical negative effects of chemo- and radiotherapy. Secondly, the type of surgery applied in these cases is generally more invasive, and could affect more input-output cerebellar pathways and cause post-surgery complications. With regard to the first point, some authors have suggested [8] that the general intellectual state could be diminished with time, and this could be associated with an impossibility for these children to acquire new skills at the same rate as control groups. In addition, there is some evidence of an association between reduced volumes of normal-appearing white matter (NAWM) and intellectual/academic deficits induced by radiotherapy [37]. Reddick [38] has demonstrated that the primary consequence of reduced NAWM in paediatric patients treated for brain tumours is a decrease in attentional abilities, leading to a decline in IQ and academic achievement.

Putting together the obtained data on IQ, motor, and visuo-motor skills and the information in the previous paragraph, the decision was taken to consider the two clinical groups as two different clinical conditions.

### Learning tasks

- Procedural learning (serial reaction-time task) [39]. The serial reaction-time task (SRT) was used to measure the implicit sequence learning. Participants were seated in front of a computer screen at a distance of 53 cm. Four open circles subtending a visual angle of 16° and arranged horizontally were presented on the screen. The dominant hand was used to respond, using four keys, on the computer keyboard, aligned with the circles. In each trial in the sequence, a circle was filled in with solid red, and the participant had to press the corresponding key. The stimulus remained filled in until a key was pressed. If the key was not pressed in one second, the trial was ended. The duration of the stimulus was 1000 ms and the ISI were randomly obtained between 500 and 800 ms. The experiment consisted of five blocks of 40 trials. We presented an additional training block before starting the real test. The sequence of red-filled circles was randomised in blocks 1 and 5. In blocks 2, 3, and 4, the stimulus was presented according to a prearranged sequence consisting of 10 stimuli which were repeated 12 times during the 3 blocks. Subjects were asked at the end of the test if they had observed any kind of sequence in the stimulus presentation. None of the children had detected the sequence. The variables obtained were (according to the type of response) hits (accuracy), errors (responses given with any other key), omissions (no response), and anticipations (correct responses with RTs faster than 250 ms), and RTs for each of the blocks independently.

- Declarative learning. (Spanish version by Benedet et al. [1]. of California Verbal Learning Test-Children's Version- CVLT- by Delis et al. [2]). This test provides variables such as learning curve, learning stability through time, short- and long-term retention, learning strategies, recovery and storage, and interference susceptibility. In our study, we focused only on the learning curve in five trials, so the variable obtained was the quantity of remembered words in each trial.

### Statistical analysis

The results were analysed using ANOVA and MANOVA tests, with the Scheffé test used for *post hoc *comparisons. The details of factors and levels are specific for each behavioural test. In order to improve the power of the analysis, all 3 "learning blocks" were included into MANOVA (block 2, 3 and 4 for the procedural task, and blocks 1 and 5 for the declarative task). The inter-subject variable was always the different clinical (CE and CE+) and control (C) groups. The normality of distributions was tested with the Shapiro-Wilk test, and the homocedasticity by means of the Levene test. As a result, the data obtained can be considered normally distributed and having similar variances (p = 0.05). For the RT analysis, when effects of the block were obtained t-tests (Bonferroni corrected for multiple comparison) were applied in order to test which block comparisons presented statistically significant differences.

## Results

### Procedural-implicit learning

**Figure 1:**
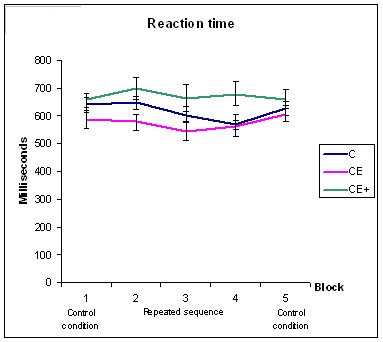
Results of groups C, CE, and CE+ during the five blocks of the sequential experiments for mean reaction times. The Standard Error of Measurement is also represented.

**Figure 2:**
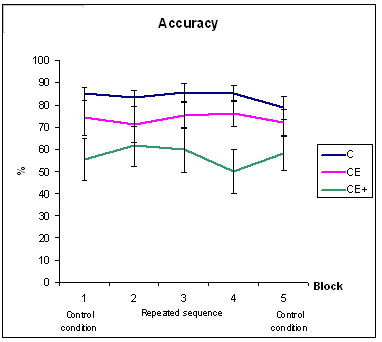
Results of groups C, CE, and CE+ during the five blocks of the sequential experiments for mean response accuracy. The Standard Error of Measurement is also represented.

Figure 1 and 2 shows the RTs and the percentage of hits respectively for the 5 experimental blocks.

Series of statistical analyses were performed separately for response accuracy and reaction times in a procedural task with two-way MANOVAs. Group (C vs. CE vs. CE+) was the between-subjects factor, while block (2 vs. 3 vs. 4) was the within-subjects factor (see Table 3 for descriptive values of these two variables).

**Table 3:** Descriptive values for reaction times and response accuracy in learning blocks from the motor sequential task. (In brackets is shown the standard deviation)

**GROUP/REACTION TIME (ms)**	**BLOCK 2**	**BLOCK 3**	**BLOCK 4**
C	647,39 (9,97)	604,35 (97,98)	568,38 (61,33)
CE	577,96 (95,34)	545,94 (109,35)	564,24 (135,50)
CE+	699,62 (106,58)	664,33 (126,39)	679,12 (116,62)

**GROUP/ACCURACY (%)**	**BLOCK 2**	**BLOCK 3**	**BLOCK 4**

C	83,12 (12,43)	85,41 (14,91)	85,00 (12,15)
CE	71,13 (26,53)	75,45 (19,22)	76,13 (18,78)
CE+	61,50 (23,38)	59,64 (26,90)	49,85 (25,98)

Analysis of the data for the factor accuracy revealed a significant effect of the group [F (2, 27) = 4.86; p = 0.016]. The *post hoc *comparisons showed that the CE+ group presented a lower number of correct responses than C (p = 0,016). No main effect of the block was obtained.

Analysis of RTs revealed significant effects of the block [F (2, 54) = 8.924; p < 0.001] and an interaction between block and group [F (4, 54) = 2.79; p = 0.035], but no main effect of the group was obtained. In order to clarify the groups where there was a change in RTs between blocks, paired t-tests (Bonferroni corrected for multiple comparisons) were performed to compare the three learning blocks for each subject group. For the C group the only obtained statistically significant differences was the block 2 with respect to block 4 (p < 0.003). However a trend to statistical significant differences were obtained when blocks 2 vs. 3 (p = 0.067) and blocks 3 vs. 4 (p = 0.095) were compared. For the CE group there was not any statistically significant differences between the different blocks, however there was a trend for statistical significance when the block 2 and 3 were compared (p = 0.064). For the CE+ group there was not any statistically significant differences between the different blocks

### Declarative-explicit learning

**Figure 3:**
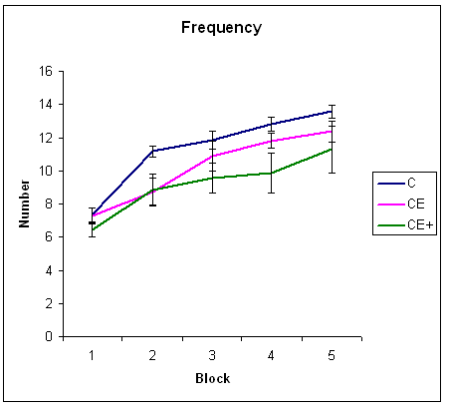
Results of groups C, CE, and CE+ during five blocks for the frequency of words remembered. The Standard Error of Measurement is also represented.

Series of statistical analyses were performed on the number of words remembered in the first and fifth blocks of the declarative test (see table 4 for descriptive values of this task). Two-way MANOVAs (repeated measurements) were used. Group (C vs. CE vs. CE+) was the between-subjects factor, while block (1 and 5) was the within-subjects factor. The analysis revealed significant effects of block [F (1, 27) = 218.99; p < 0.001] nor effects of group nor interaction between the effects of the block and group were found. Figure 3 displays the overall results in the five blocks of the declarative tasks.

**Table 4:** Descriptive values for frequency of remembered words in learning blocks from the declarative-verbal task. (In brackets is shown the standard deviation)

**GROUP/FREQUENCY**	**BLOCK 1**	**BLOCK 5**
C	7,33 (1,43)	13,58 (1,37)
CE	7,27 (1,55)	12,36 (2,06)
CE+	6,42 (1,13)	11,28 (3,72)

**Figure 4:**
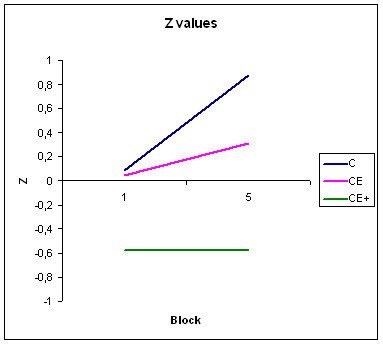
Z-values for groups C, CE, and CE+ in the first and fifth blocks of the declarative learning test. The displayed Z-values are compared to a Spanish "norm"

Finally, figure 4 shows the z-values using the normalised tables for a Spanish population in the first and fifth blocks of the declarative learning test. All the groups were within the normal population range (± 1 S.D.), but group CE+ was consistently under the mean value for the normalised population.

## Discussion

The present study was designed to assess procedural vs. declarative learning in children and adolescents with acquired pathology confined to the cerebellum (CE), and in children and adolescents with additional damage due to the chemotherapy and radiotherapy used (CE+). The characteristics of the clinical subjects included in the study did not show a major impairment of subjects that prevented their inclusion in the experiment. It should be noted that the conservation of basic motor skills, and of visuomotor and cognitive functioning of the two clinical groups, allows the testing of declarative and procedural learning.

Our results show differential alterations in learning capacity in children with posterior fossa tumours. The ANOVA of procedural data show that neither of the clinical groups was capable of learning motor sequences in spite of having sufficient basic visuo-motor skills to complete the task. The differences between clinical groups and controls could be explained by the involvement of cerebellar circuits in procedural learning. On the other hand, no statistical differences were found in the task related with declarative memory. The fact that the CE+ group showed a trend – non-statistically significant – towards being more affected than the CE group could be due to different factors such as the greater malignancy, the type of surgery employed, the post-surgical outcome, and coadjuvant treatments.

Our results support the broad evidence favouring the involvement of the cerebellum in procedural learning. There are many studies that indicate subcortical involvement in procedural learning, suggesting different roles for basal ganglia and cerebellum. Numerous studies with patients showing cerebellar disease or striatum degenerative disease support the relationship between these structures and procedural learning [40-44]. Gómez-Beldarrain et al. [45] have proposed functional cerebellar lateralised deficits due to cerebellar focal lesions. Children with neurodevelopmental disorders involving fronto-cerebellar lesions (e.g., autism) have also been shown to be impaired in procedural learning [21]. In the latter two studies, a serial reaction-time task was used, as opposed to some other studies using more-clinically oriented tests. Daum [46] used two procedural learning tasks involving perceptual (mirror reading) and conceptual (Tower of Hanoi task) skill acquisition, and found that patients with cerebellar pathology showed no effect on skill acquisition. Daum's results could be interpreted as being that if more cognitive operations are involved in the two proposed tasks, then the procedural operations in the tasks could be compensated by cognitive functions. In our experiment, subjects were not aware of the hidden sequence, so that cognitive operations would have great difficulty in compensating the deficits in the serial task.

In line with these earlier studies showing that procedural learning is severely impaired in patients with different kinds of cerebellar damage are the experiments using positron emission tomography (PET), in which changes in cerebral metabolic activity have been observed in the striatum and the cerebellum during procedural learning [47-49]. In the same sense, it has been demonstrated that the interference of cerebellar activity by transcranial magnetic stimulation produces an interference of procedural learning of a serial sequence task [50].

Some recent studies using PET and fMRI have been interested in identifying the neural networks involved in different stages of sequential learning [51]. A revised neurocognitive model of learning new sequences of motor control [52], based on a previous model [53], proposed that in the fast learning phase that occurs in motor learning during the first blocks of trials there is an involvement of cerebellar and striatal circuits. The learning progresses to a specialisation of the cerebellum in adaptation tasks, and a specialisation of striatal circuits in sequence learning. Present results of impaired sequential procedural learning support the involvement of the cerebellum in the early phase of sequential motor learning. However, the fact that the CE group presented a statistically significant trend for procedural learning when blocks 2 and 3 are compared suggest that, as proposed by current models of procedural learning [52,53], the striatal circuit would play the most important role in sequential learning.

With regard to declarative learning, effective learning occurred in the two clinical groups. No interaction appeared between the effects of learning block and group. However, qualitatively it can be appreciated that CE+ performance was the lower, particularly in z-scores, but learning still remained within the score limits for a normal population.

The role of the cerebellum in declarative learning has been proved in tasks where visual memories must be formed and retrieved [54]. More similarly to our study (because the encoded items were verbal), there are some neuroimaging studies that show an increase of activation in the cerebellum during memory tasks involving the encoding of sentences [55] or of auditory-verbal material [56]. However, as far as we know, the only experiment that tried to find the areas correlated with the California Verbal Memory Test (similar to our declarative learning task) did not show an activation in the cerebellum [57]. In the present report, in accord with other results, declarative memory is preserved in these clinical groups. In some studies that assess declarative learning from the sequence used to assess procedural learning [58], this kind of memory is conserved in adult clinical samples. By contrast, Parkinsonian patients and patients with cerebellar lesions failed to improve task performance when only procedural processes were used.

## Conclusion

Our results can support the hypothesis of cerebellar involvement in the early phases of procedural learning acquisition [46,52]. On the other hand, the early verbal declarative learning system was preserved. However, the cerebellum's role in motor learning [59] – but also in cognitive functions [60] – could be in reinforcement of the process rather than in the process itself. Thus it is possible that, with enough number of trials, procedural learning could still occur in cerebellar patients.

## Competing interests

The author(s) declare that they have no competing interests.

## Authors' contributions

EAQG participated in the design of the study, collected the clinical neuropsychological data, and helped to draft the manuscript. CMG conceived the study, participated in its design, and drafted the manuscript. EVC participated in the drafting and analysis. JM helped to select the participants in the study, and medically characterised each one of the subjects. FJPS performed the statistical analysis. All authors read and approved the final manuscript.
